# Deciphering the mode of action of cell wall-inhibiting antibiotics using metabolic labeling of growing peptidoglycan in *Streptococcus pyogenes*

**DOI:** 10.1038/s41598-017-01267-5

**Published:** 2017-04-25

**Authors:** Atsushi Sugimoto, Asuka Maeda, Kaori Itto, Hirokazu Arimoto

**Affiliations:** Graduate School of Life Sciences, Tohoku University, Sendai, Miyagi Japan

## Abstract

Because of the scanty pipeline of antibiotics newly obtained from nature, chemical modification of established drugs is one of the major streams of current antibacterial research. Intuitive and easy-to-use assays are critical for identifying drug candidates with novel modes of action. In this study, we demonstrated that metabolic fluorescent staining of growing cell walls is a powerful tool for mode-of-action analyses of antibiotics using *Streptococcus pyogenes*. A set of major cell-wall-inhibiting antibiotics (bacitracin, d-cycloserine, flavomycin, oxacillin, ramoplanin, and vancomycin) was employed to validate the potential of the assay. The mechanistic differences of these antibiotics were successfully observed. For instance, d-cycloserine treatment induced fluorescently stained, excessive peripheral cell wall growth. This may indicate that the switch from the peripheral growth stage to the succeeding septal growth was disturbed by the treatment. We then applied this assay to analyze a series of vancomycin derivatives. The assay was sufficiently sensitive to detect the effects of single-site chemical modification of vancomycin on its modes of action. This metabolic fluorescent labeling method is easy to perform, especially because it does not require radiolabeled substrates. Thus, it is suitable for the preliminary evaluation of antibacterial mechanisms during antibacterial research.

## Introduction

Antibiotic resistance is a global challenge, and effective strategies for identifying novel antibacterial compounds with promising modes of action are in great demand. The minimum inhibitory concentration (MIC) is an essential criterion for screening large compound libraries or microbial extracts in the pursuit of new antibacterial agents, but it does not provide insight into the modes of action. Therefore, the incorporation of radiolabeled precursors into biomacromolecules, e.g., peptidoglycan, nucleotides, and proteins, is often examined to roughly identify the targets of antibiotics.

Cell wall biosynthesis is a major target of antibiotics, including beta-lactam and glycopeptide antibiotics. *In vitro* reconstitution of peptidoglycan biosynthesis using semi- or completely purified enzymes and radiolabeled precursors has been utilized to analyze the action of cell wall-inhibiting antibiotics at the molecular level. A major drawback of this *in vitro* assay is a limited supply of radiolabeled precursors, specifically those for late-stage peptidoglycan synthesis. Chemical synthesis is currently the most practical means of supplying these key precursors, but it often requires years of laborious synthetic efforts to provide several milligrams of precursors. In this context, we recently reported the first chemical synthesis of depsi-lipid intermediates of vancomycin-resistant strains^1^. Another concern regarding the reconstituted assay using a purified enzyme is that the system may not reflect the true situation of cell wall biosynthesis as orchestrated by the dynamic interplay among multiple enzymes.

We envisaged that whole cell-based assays could compensate for the drawbacks of the *in vitro* enzyme-based assay. To monitor the actions of cell wall-inhibiting antibiotics, efficient labeling methods for newly forming cell walls are needed. Recently, Nelson *et al*. used endogenous sortase to label living *Staphylococcus aureus* ^2^. Sortase recognizes the specific C-terminal sequence of cell wall-binding proteins and connects them with the cell wall precursor lipid II^3^. By employing an artificial sortase-substrate with a fluorescent chromophore, microscopic observation of newly synthesized *S*. *aureus* cell walls was successfully demonstrated^2^. However, the application of this strategy for living cells has been limited to *S*. *aureus* ^4^ because sortases have different substrate specificities among bacteria.

In this study, we demonstrated that metabolic labeling can be a powerful and quick means to obtain insight into the modes of action of new antibacterial compounds. This imaging-based assay with group A streptococcus (GAS, *Streptococcus pyogenes*) was able to distinguish the actions of six major cell wall-inhibiting antibiotics together with a series of structurally similar vancomycin derivatives.

## Results

### Live monitoring of S. pyogenes cell wall biosynthesis using sortase-mediated metabolic labeling

**Figure 1:**
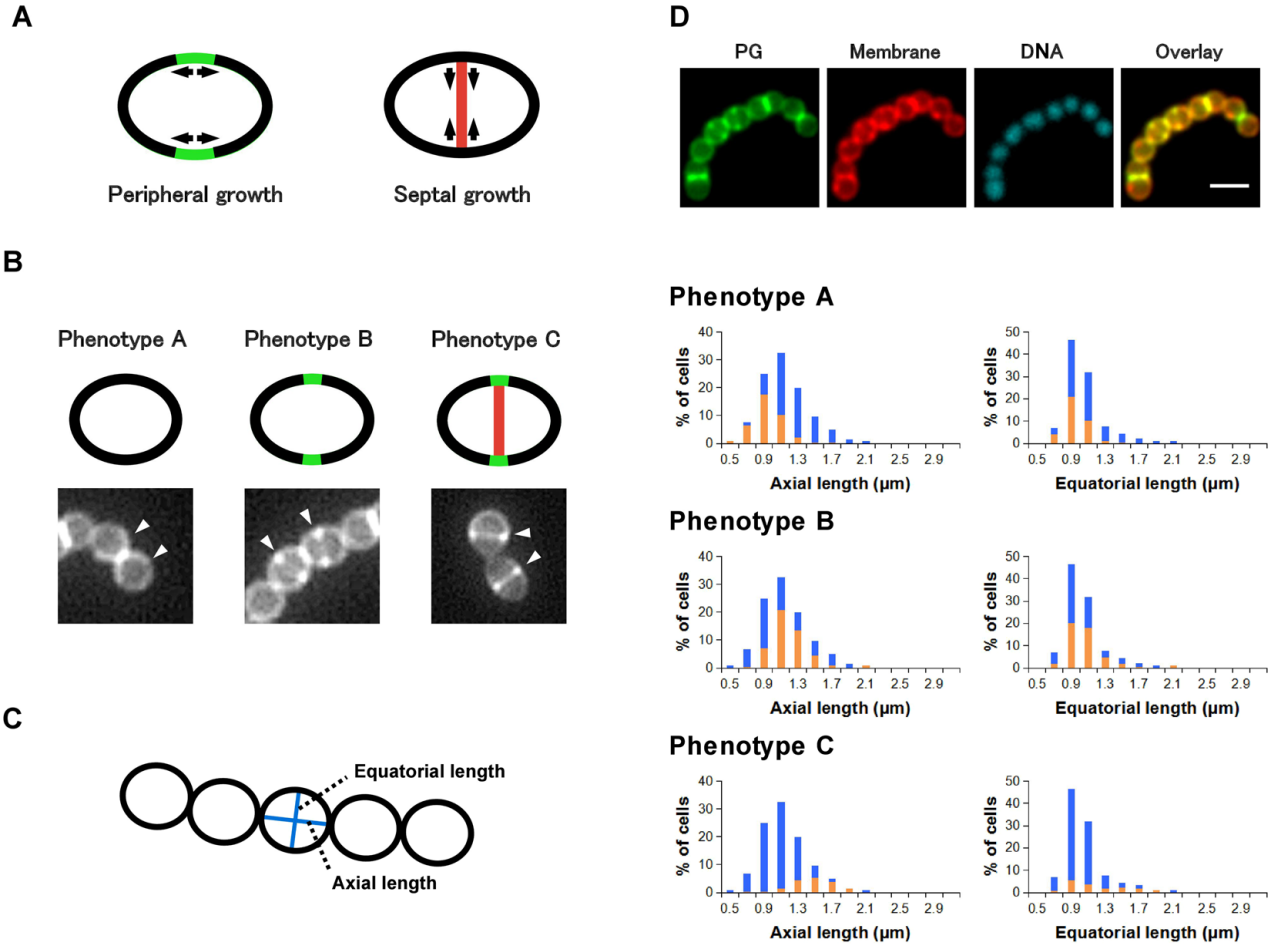
Distribution of cell sizes and phenotypes of log-phase *Streptococcus pyogenes* in the absence of antibiotics. (**A**) Schematic presentations of peripheral and septal cell wall synthesis in *Streptococcus*. (**B**) Typical labeling patterns in metabolic labeling of *Streptococcus pyogenes* cell wall synthesis using a fluorescent sortase A substrate. Phenotype A: cells without active cell wall synthesis. Phenotype B: cells undergoing peripheral growth. Phenotype C: cells undergoing septal growth. Each phenotype reflects the bacterial cell cycle. (**C**) Definition of axial and equatorial length in this study. (**D**) Typical fluorescent images and the average cell-size distribution of phenotypes A–C (n = 3). Images: PG (growing peptidoglycan stained using the sortase method), Membrane (Nile red staining), DNA (DAPI), and Overlay (overlay of PG and Membrane images); scale bar: 2 μm. Blue bars in histograms represent the cell size distribution of all cells. Orange bars in histograms represent the size distribution cells with the indicated phenotypes. Statistical analysis regarding the histograms is available in Supplementary Table S1.

GAS causes a wide variety of diseases in humans. The first important step toward a new imaging-based assay was the optimization of labeling conditions because no study had examined labeling of the GAS cell wall by the sortase method before. Sortase A was known to accept peptides with a C-terminal LPXTG sequence in both GAS and *S*. *aureus*, and form an intermediate that is subsequently linked to peptidoglycan. We found that sufficient labeling of a mid-log phase GAS proceeded within 1 h with the fluorescent substrate FL-KLPETG-NH_2_ while using the sortase method, whereas it required 12 h with *S*. *aureus* originally used by Nelson *et al*.^2^. Regarding live cell monitoring of cell wall biosynthesis, the observed large difference in labeling efficiency is noteworthy because labeling should be as fast as possible. Thus, we decided to use the GAS JRS4 strain and sortase-mediated labeling for our study (Fig. 1).

Streptococcal cell wall synthesis consists of cylindrical peripheral synthesis and septal synthesis (Fig. 1A). A study using *Streptococcus pneumoniae* illustrated that the serine-threonine kinase StkP controls the switch from peripheral synthesis to septal synthesis^5^. Splitting of the septum (cell separation) is then mediated by the action of autolysin. We labeled the GAS cell wall using the sortase method in the absence of antibiotics, and the observed labeling patterns of GAS were classified into three phenotypes (Fig. 1A–C). Phenotype A cells are newly divided cells without specific fluorescent labeling. Phenotype B cells are in the peripheral growth stage. A characteristic two-elongated-dot image or an open ring corresponds to peripheral growth near the bacterial division site. Phenotype C cells are in the septal growth stage, and the dividing septum is fluorescently stained. The distribution (%) of phenotypes A, B, and C among cells was 37 ± 2, 47 ± 2, and 16 ± 1, respectively, in log-phase GAS. Data represent the mean ± sem (n = 3). Subsequently, we constructed a histogram of each phenotype population as a function of bacterial cell length, as defined in Fig. 1C (orange, Fig. 1D). The subpopulation of cells with a specific phenotype is overlaid on the total cell size distribution (blue). The histograms suggested that GAS elongates mostly along the axial direction in the progression from phenotype A to phenotype C, and growth along the equatorial direction is smaller. The histograms also illustrate that cells grow from phenotype A, through phenotype B, to phenotype C (followed by cell separation), confirming that peripheral growth precedes septal growth in GAS. We speculated that the changes of this histogram would provide information on antibiotic modes of action.

### Histogram analyses of cell size and phenotypes in the presence of cell wall-inhibiting antibiotics

We then performed similar histogram analyses in the presence of cell wall-inhibiting antibiotics namely bacitracin, flavomycin, d-cycloserine, oxacillin, and ramoplanin. Because we employed these drugs at their subbacteriostatic concentrations, metabolic-fluorescent labeling could proceed slowly in living cells (see the Materials and methods section for determination of subbacteriostatic concentration for each antibiotic). Although all of these antibiotics are known to inhibit peptidoglycan synthesis, the observed abnormalities in bacterial size and shape varied among the antibiotic treatments. These results may be due to the differences in the stages of cell wall biosynthesis inhibited by the compounds.

### Bacitracin and ramoplanin halted cell wall growth and reduced the size of GAS cells

**Figure 2:**
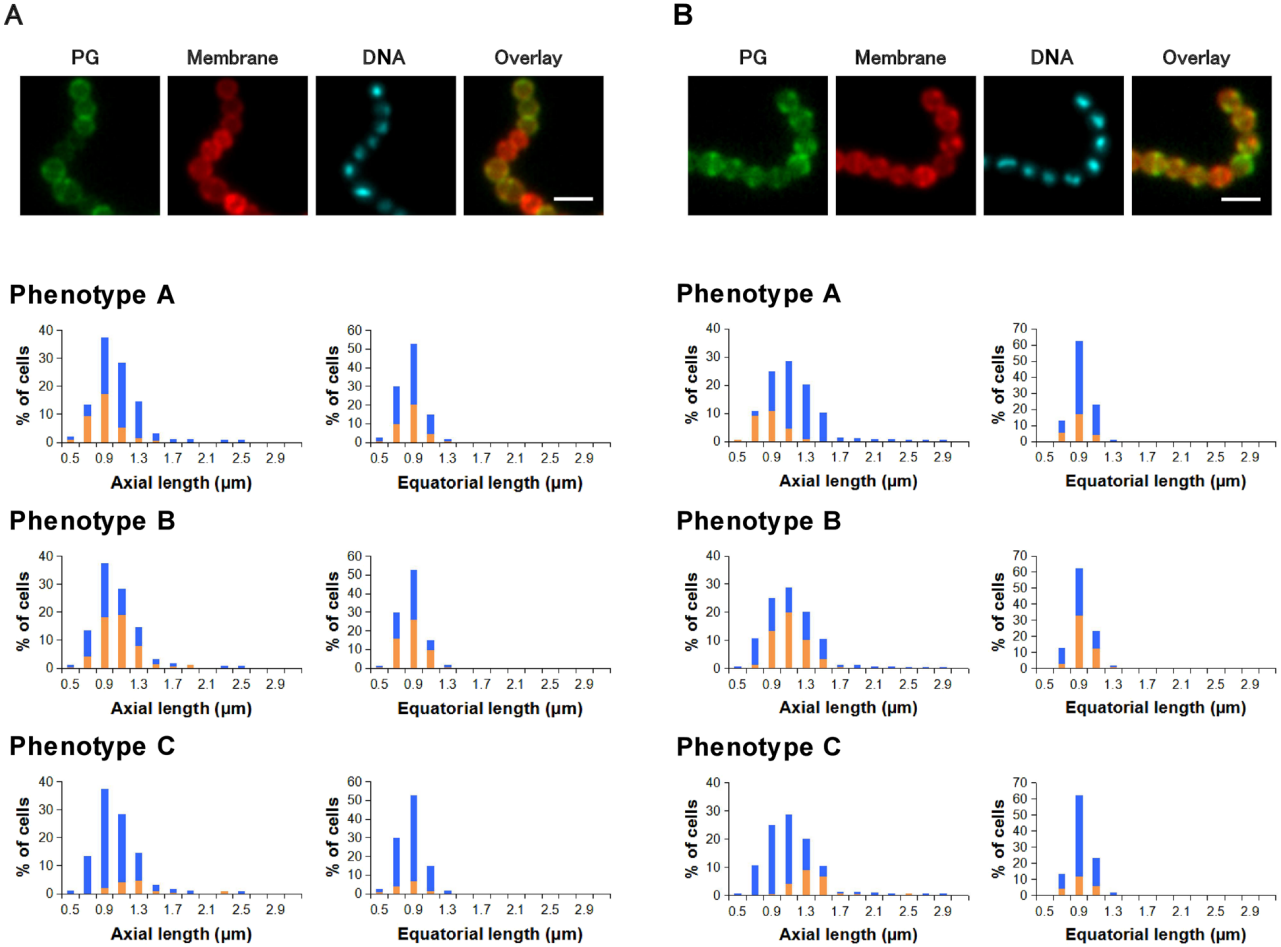
Average distribution of cell sizes and phenotypes of bacitracin (4 μg/mL)-treated *Streptococcus pyogenes* (**A**) and ramoplanin (2 μg/mL)-treated *S*. *pyogenes* (**B**) (n = 3). Images: PG (growing peptidoglycan stained using the sortase method), Membrane (Nile red staining), DNA (DAPI), and Overlay (overlay of PG and Membrane images); scale bar: 2 μm. Blue bars in histograms represent the cell size distribution of all cells. Orange bars in histograms represent the size distribution cells with the indicated phenotypes. Statistical analysis regarding the histograms is available in Supplementary Table S2.

Bacitracin suppresses the formation of late-stage peptidoglycan intermediates (lipid intermediates) by inhibiting lipid phosphorylase. These lipid intermediates are used in both peripheral and septal growth. In the bacitracin-treated bacteria, the distribution (%) of each phenotype among the cells was 31 ± 5, 52 ± 5, and 12 ± 0 for phenotypes A, B, and C respectively (n = 3). A slight accumulation of phenotype-B cells (peripheral growth stage) was observed, but the overall distribution was similar to that of non-treated cells. A rapid decline of essential substrates by bacitracin treatment might halt each stage of cell wall synthesis. Bacitracin was also found to reduce both axial and equatorial length distributions (Fig. 2A) compared with the findings in non-treated cells (Fig. 1).

Ramoplanin is a lipopeptide antibiotic that inhibits the intracellular glycosyltransferase MurG which converts lipid I to lipid II via the addition of an *N*-acetylglucosamine. As the outcome of this effect, ramoplanin can also suppress the formation of essential lipid intermediates for cell wall synthesis. In the ramoplanin-treated bacteria (Fig. 2B), the average distribution (%) of each phenotype among cells was 27 ± 3 (phenotype A), 50 ± 4 (phenotype B), and 23 ± 5 (phenotype C) (n = 3). The distribution was similar to that of bacitracin, and this may reflect the modes of action of these antibiotics.

Although the specific actions of ramoplanin on *S*. *pyogenes* are not fully known, a previous study reported using purified *E*. *coli* MurG and *E*. *coli* PBP1b, the major transglycosylase in the organism, and the transglycosylation step was found to be preferentially blocked compared with the MurG step^6^. Thus, we were interested in whether this second action of ramoplanin (inhibition of cell wall polymerization) resulted in a different phenotype distribution from that of bacitracin. However, the results suggested that ramoplanin acts mainly as an inhibitor of lipid intermediate formation in *S*. *pyogenes* at subbacteriostatic concentration.

### Flavomycin inhibited cell separation

**Figure 3:**
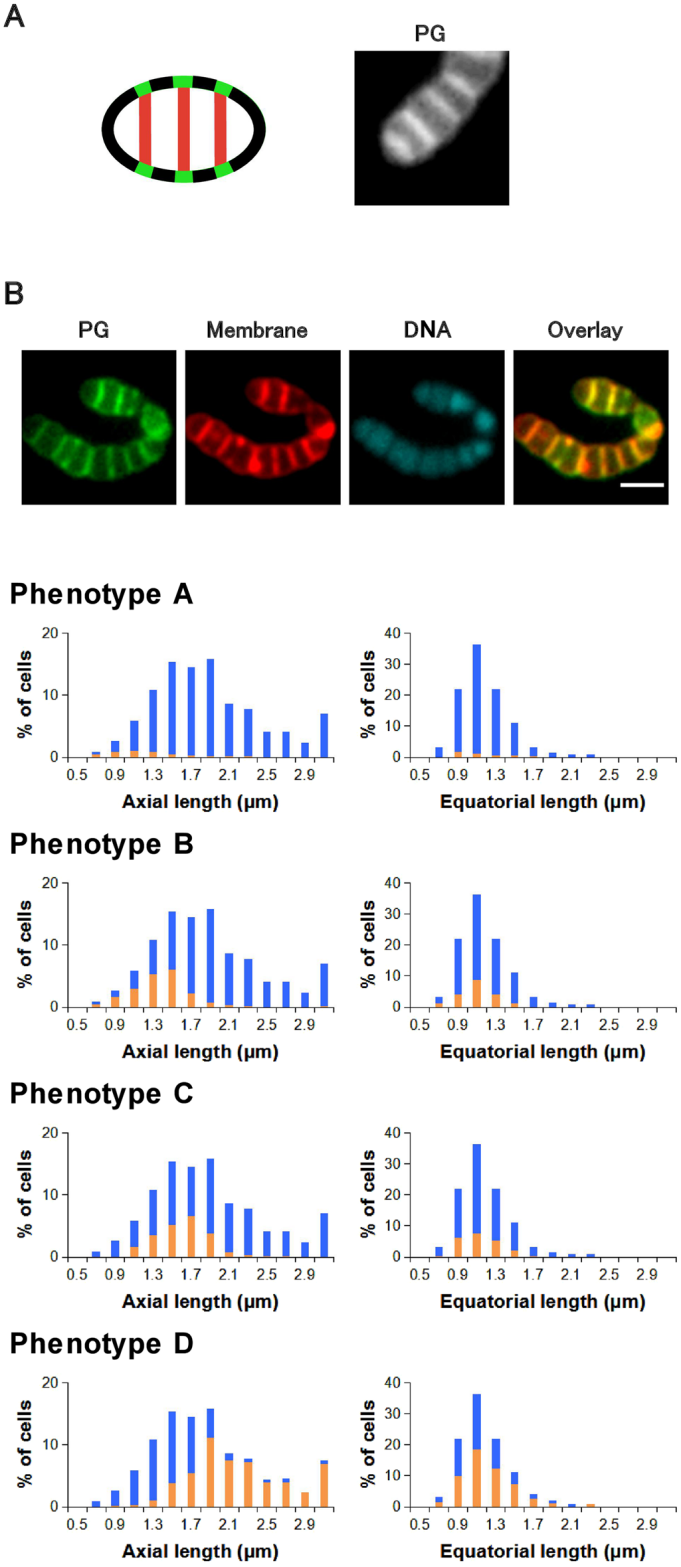
Average distribution of cell sizes and phenotypes of flavomycin (also known as moenomycin) (1 μg/mL)-treated *Streptococcus pyogenes* (n = 3). (**A**) Phenotype D represents cells with multiple septal growths (left: schematic presentation of phenotype D in flavomycin-treated *S*. *pyogenes*; right: microscopic image of a phenotype-D cell stained using the sortase method). (**B**) Typical fluorescent images and cell size distributions of phenotypes A–D. Images: PG (growing peptidoglycan stained using the sortase method), Membrane (Nile red staining), DNA (DAPI), and Overlay (overlay of PG and Membrane images); scale bar: 2 μm. Blue bars in histograms represent the cell size distribution of all cells. Orange bars in histograms represent the size distribution of cells with the indicated phenotypes. Statistical analysis regarding the histograms is available in Supplementary Table S3.

Flavomycin is a glycolipid antibiotic that suppresses peptidoglycan polymerization by inhibiting transglycosylase. In the flavomycin-treated bacteria (Fig. 3), the average proportions (%) of the phenotypes among the cells was 4 ± 1 (phenotype A), 20 ± 6 (phenotype B), 22 ± 2 (phenotype C), and 54 ± 5 (phenotype D) (n = 3). The predominant phenotype (D), which was not observed under normal conditions, had multiple septa in an elongated cell (Fig. 3A). The elongated cell with several septa could also be regarded as a cell cluster with defective cell separation, but we treated this phenotype D as a single cell that had not completed a normal cell division cycle in this study. The cell separation step is a necessary step for normal growth. Labeling of these multiple septa suggested that septal growth proceeded in the presence of flavomycin at subbacteriostatic concentration. The accumulation of phenotype D thus would imply that flavomycin selectively stopped the cell separation step of *S*. *pyogenes* and that the subsequent peripheral and septal cell wall synthesis could proceed regardless of the failure of the preceding cell separation step. This process may eventually provide an additional septum in an elongated cell. The low population of phenotype A also supported this interpretation because it implied the failure of normal cell division in most growing cells. As flavomycin is considered an inhibitor of the transglycosylation step of peptidoglycan polymerization mediated by penicillin-binding proteins (PBPs), these results (inhibition of cell separation rather than cell wall synthesis) at subbacteriostatic concentration were unexpected. In fact, the inhibitory effect of flavomycin on cell separation was reported recently for *S*. *aureus* ^7^, but to our knowledge, it has not been well described for *S*. *pyogenes*. Strepcococci use multiple enzymes (PBP1A, PBP1B, and PBP2A) for peptidoglycan polymerization^8^. An interesting future study would involve identification of specific PBPs responsible for the defect of cell separation.

### GAS exhibited enlarged cell size and phenotype B was accumulated after d-cycloserine or oxacillin treatment

d-Cycloserine is a cyclic analog of d-alanine that blocks the early cytoplasmic phase of peptidoglycan synthesis by inhibiting d-alanyl-d-alanyl ligase (Ddl). Thus, d-cycloserine treatment produces abnormal tripeptidic cell wall precursors (vs normal pentapeptidic precursors)^9^. By contrast, oxacillin blocks the late stage of peptidoglycan biosynthesis by inhibiting the action of PBPs that cross-link the immature peptidoglycan. It is intriguing to observe that these mechanistically different inhibitors induced apparently similar enlarged cell morphology.

**Figure 4:**
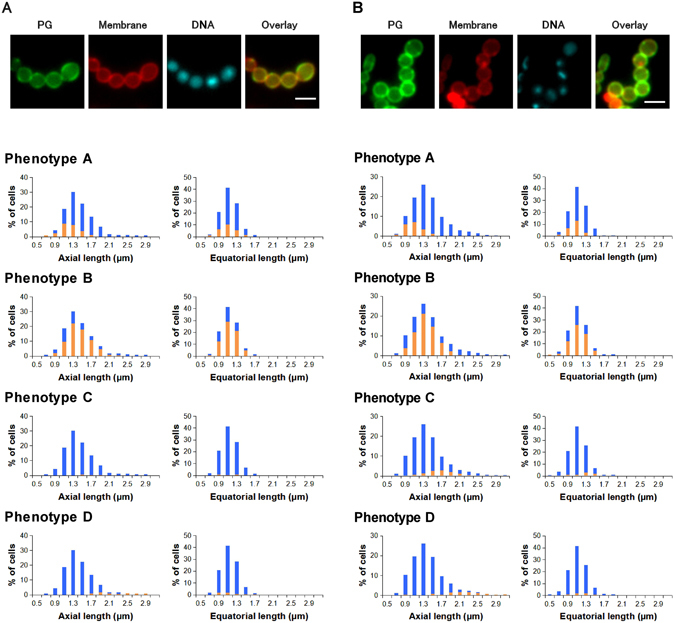
Average distribution of cell sizes and phenotypes of d -cycloserine (100 μg/mL)-treated *Streptococcus pyogenes* (**A**) and oxacillin (4 μg/mL)-treated *S*. *pyogenes* (**B**) (n = 3). Images: PG (growing peptidoglycan stained using the sortase method), Membrane (Nile red staining), DNA (DAPI), and Overlay (overlay of PG and Membrane images); scale bar: 2 μm. Blue bars in histograms represent the cell size distribution of all cells. Orange bars in histograms represent the size distribution cells with the indicated phenotypes. Statistical analysis regarding the histograms is available in Supplementary Table S4.

In the d-cycloserine-treated bacteria (Fig. 4A), the average proportions (%) of the phenotypes among cells were 25 ± 6 (phenotype A), 68 ± 7 (phenotype B), 2 ± 1 (phenotype C), and 5 ± 1 (phenotype D) in three independent experiments. The accumulation of cells staying at the peripheral growth stage (phenotype B) was therefore characteristic. Spherical enlarged cells, in which both axial and equatorial lengths increased, were observed for most phenotype-B cells (Fig. 4A). Conversely, cells at the septal growth stage (phenotype C) were scarce. The abnormal tripeptidic precursors might inhibit switching from the peripheral (phenotype B) to the septal growth stage (phenotype C). As previously mentioned, the streptococci serine-threonine kinase StkP regulates the switch from peripheral synthesis to septal synthesis^5^. The phenotype-D population, which was predominant in the flavomycin-treated cells, was small (5%) under these conditions.

In the oxacillin-treated bacteria (Fig. 4B), the average proportions (%) of each phenotype among the cells were 19 ± 4 (phenotype A), 60 ± 4 (phenotype B), 12 ± 5 (phenotype C), and 9 ± 2 (phenotype D) (n = 3). The accumulation of phenotype-B cells and enlargement of cells were also observed in the presence of oxacillin. Although the cell size distributions of the d-cycloserine- and oxacillin-treated cells (blue, Fig. 4A and B) were similar, we further investigated the differences of mechanisms of the two antibiotics other than the abnormal enlarged morphology in the following section.

### Imaging of peripheral cell wall growth revealed the differences of d-cycloserine and oxacillin concerning the mechanism of cell enlargement

**Figure 5:**
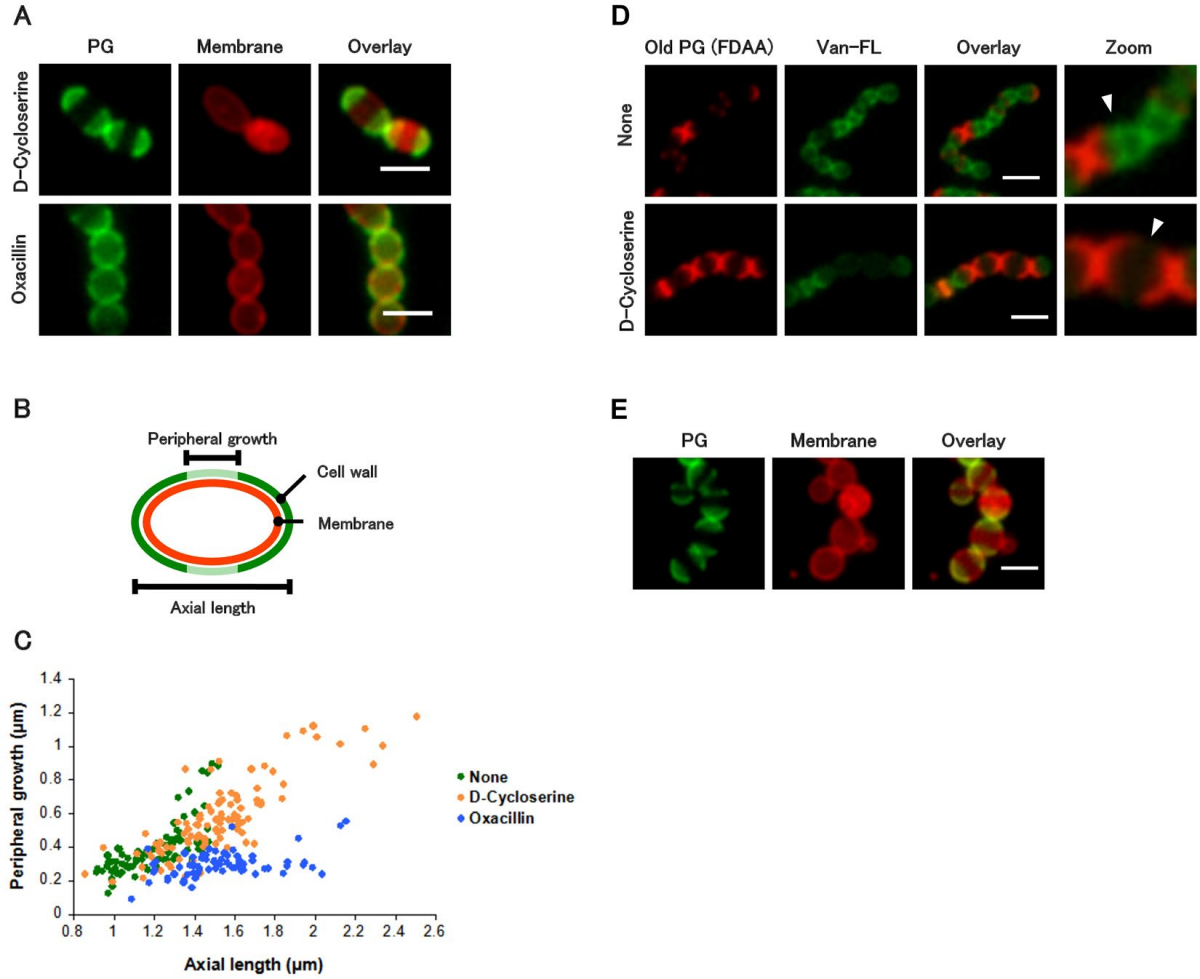
Excessive peripheral growth is the major cause of cell enlargement in d-cycloserine-treated cells, whereas peripheral growth has little contribution to cell size in oxacillin-treated cells. (**A**) *Streptococcus pyogenes* was pre-labeled with FDAA (1 h) and then allowed to grow without FDAA in the presence of d-cycloserine/oxacillin (3 h). scale bar: 2 μm. (**B**) Schematic presentation of peripheral and axial lengths in these experiments. The length of peripheral growth was estimated as the gap between old (stained) cell wall regions. (**C**) A dot blot analysis of the peripheral-growth/axial-length relationships of each cell suggested that peripheral growth barely contributed to cell enlargement in oxacillin-treated cells. Cells were treated with antibiotics for 3 h. Non-treated cells were analyzed 1 h after the FDAA pre-labeling because of their rapid growth. (**D**) Van-FL was used to stain cell walls with d-Ala-d-Ala terminus (30 min prior to analysis). The peripheral wall is expected to exist at a gap between old peptidoglycan regions. Van-FL staining did not localize at the newly synthesized peripheral cell wall (white arrow heads) in the presence of d-cycloserine. (**E**) Cell lysis in the presence of d-cycloserine at an excessive peripheral growth region; scale bar: 2 μm.

The observed enlargement of cells implied that cell wall synthesis could proceed, at least partly, in the presence of these antibiotics at their subbacteriostatic concentrations. We therefore studied the relationship between peripheral growth length and axial length for cells via dual-color staining. Nile red was used to stain the bacterial cell membrane, and the fluorescent d-amino acid (FDAA) labeling^10–12^ was employed for old cell wall (preexisting prior to antibiotic treatment). In the presence of antibiotic, peripheral growth length was measured as a gap between the old (stained) regions of the preexisting cell wall (Fig. 5A and B).

As depicted in Fig. 4, both d-cycloserine- and oxacillin-treated cells had similar cell size distributions. However, a dot blot analysis (Fig. 5C) demonstrated that the peripheral growth/axial length ratio of d-cycloserine-treated cells was considerably larger than that of oxacillin-treated cells. This result indicated that enlargement of d-cycloserine-treated cells was the result of continued excessive peripheral growth, which may have been due to suppression of the switch from peripheral to septal growth. We then examined whether the region of the excessive peripheral growth lacked the d-Ala- d-Ala peptide using Van-FL staining (Fig. 5D). Van-FL, a fluorescent vancomycin derivative, has been used for detecting the d-Ala- d-Ala substructure because of its specific binding to this peptide. This indicates that this region was created from the abnormal tripeptidic precursors. Because the d-Ala- d-Ala sequence is essential for the cross-linking (transpeptidation) of immature peptidoglycan, the region may lack sufficient strength as a cell wall. Accordingly, site-specific bulge formation was often observed in the d-cycloserine-treated *S*. *pyogenes* (Fig. 5E).

In contrast to the results of d-cycloserine treatment, the peripheral growth length could not explain the increased size of oxacillin-treated cells as assessed by dot blot analysis. Cell enlargement induced by oxacillin should be mediated by the growth of the remaining preexisting region (presumably the outer wall region). In *S*. *aureus*, oxacillin was found to delocalize PBP2, a major *S*. *aureus* enzyme responsible for peptidoglycan polymerization, over the entire surface of the cell^13^. Similar PBP delocalization may also be the cause of un-orchestrated cell wall synthesis in the outer wall region of *S*. *pyogenes*.

### Application of cell wall labeling for mechanistic analyses of vancomycin and its semi-synthetic derivatives CBPV and ΔNCBPV

**Figure 6:**
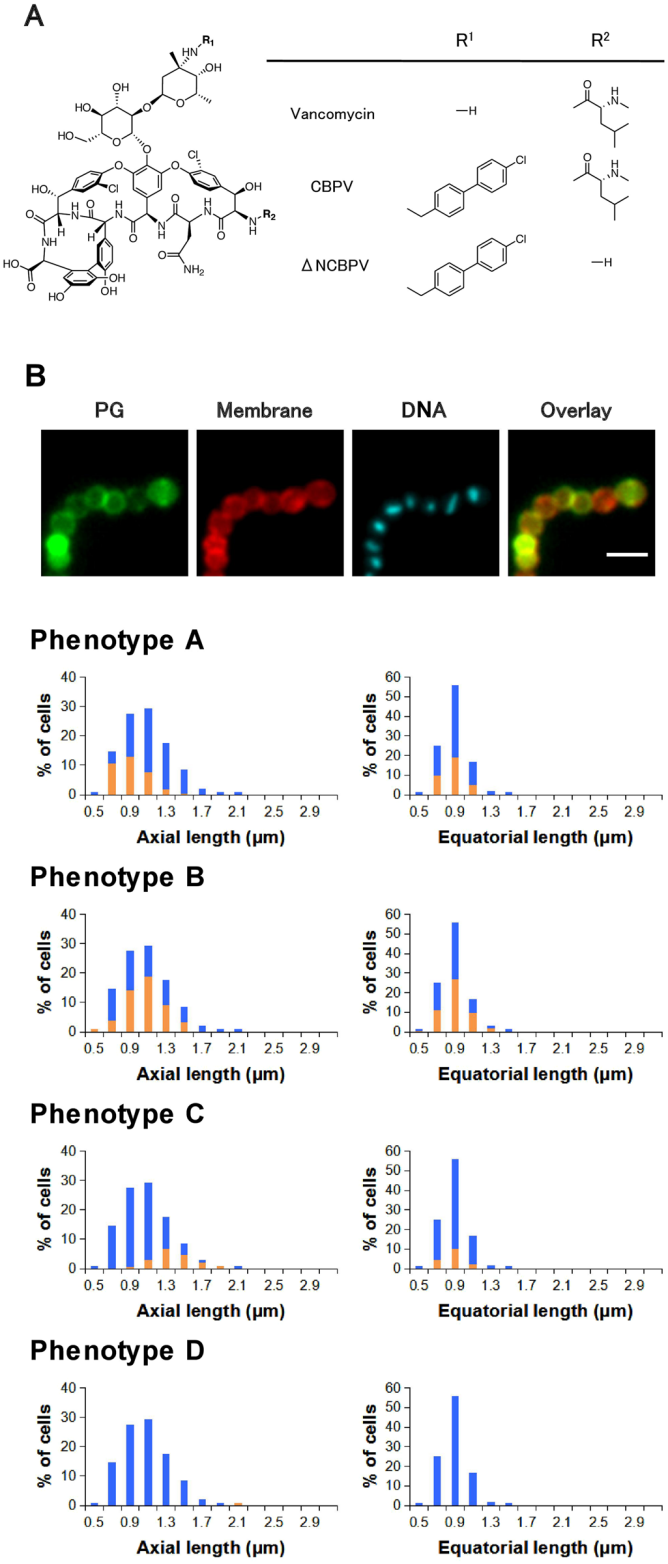
Average distributions of cell sizes and phenotypes of vancomycin (4 μg/mL)-treated *Streptococcus pyogenes* (n = 3). (**A**) Chemical structures of vancomycin, chlorobiphenyl vancomycin (CBPV), and des-*N-*methylleucyl CBPV (ΔNCBPV). (**B**) Typical fluorescent images and cell size distribution of phenotypes A–C in the presence of vancomycin. Images: PG (growing peptidoglycan was stained using the sortase method), Membrane (Nile red staining), DNA (DAPI), and Overlay (overlay of PG and Membrane images); scale bar: 2 μm. Blue bars in histograms represent the cell size distribution of all cells. Orange bars in histograms represent the size distribution of cells with the indicated phenotypes. Statistical analysis regarding the histograms is available in Supplementary Table S5.

Vancomycin is the drug of choice for treating infections caused by multi-resistant gram-positive pathogens, including MRSA. CBPV is a synthetic derivative of vancomycin with a chlorobiphenylmethyl modification at the sugar moiety of vancomycin (Fig. 6A)^14^. Oritavancin, which also possesses a chlorobiphenylmethyl substituent was recently approved by US Food and Drug Administration (FDA) as antibacterial agent. These lipoglycopeptides display excellent activity against vancomycin-resistant strains, and the mechanism of the enhanced activity has been the subject of other studies^15, 16^. We applied our imaging-based analysis to obtain further insights into the mechanisms of these vancomycin derivatives.

We first examined the effect of unmodified vancomycin on GAS morphology and cell cycle progression. Vancomycin exerts its antimicrobial activity via a specific interaction with the d-Ala-d-Ala terminus of cell wall intermediates on the bacterial surface. This mechanism is unique among the antibiotics used in this study, as only vancomycin targets the enzymatic substrate opposed to the enzyme itself. We found that vancomycin (4 μg/mL) inhibited the growth of GAS, but the cell size distribution was not affected along the axial and equatorial directions (Fig. 6B). The cell size distribution was similar to that in the absence of antibiotics at the log phase (Fig. 1). Moreover, the distribution of phenotypes A–C, which reflected the cell cycle stage of each cell, was also least affected. The proportions (%) were 34 ± 4 (phenotype A), 49 ± 4 (phenotype B), and 17 ± 1 (phenotype C) (n = 3). The results were similar to those for bacitracin and ramoplanin but distinct from those with other cell wall-inhibiting antibiotics used in this study (e.g., d-cycloserine, flavomycin, and oxacillin). This can be rationalized because all of these three antibiotics reduce the levels of the precursors available for cell wall synthesis. Bacitracin and ramoplanin are established inhibitors of cell wall precursor biosynthesis, whereas vancomycin strongly binds to the precursors to sterically suppress their use for enzymatic reactions. Regardless of these differences, the overall outcome is the depletion of cell wall precursors.

**Figure 7:**
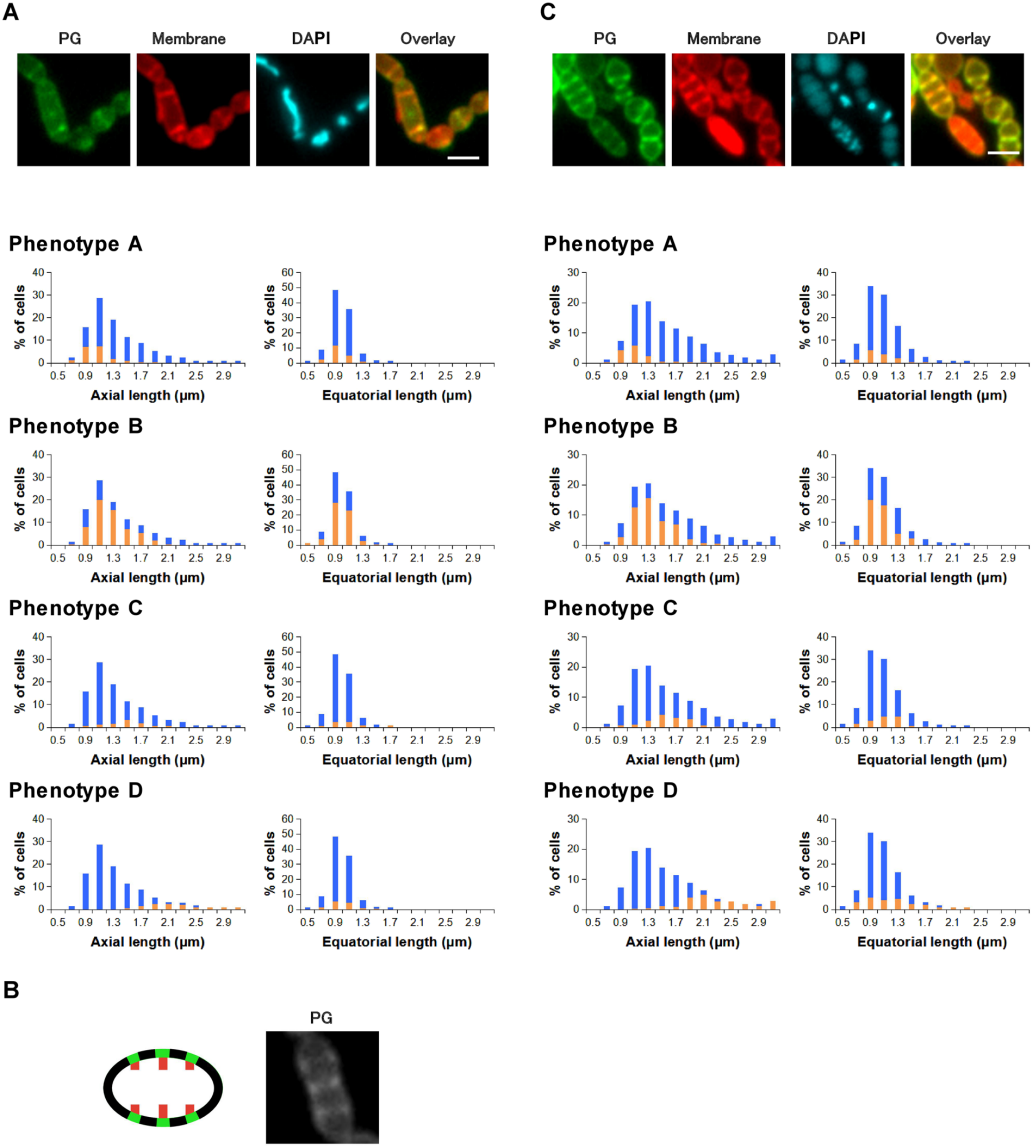
Average distributions of cell sizes and phenotypes of chlorobiphenyl vancomycin (0.25 μg/mL)-treated *Streptococcus pyogenes* (**A**,**B**) and des-*N-*methylleucyl chlorobiphenyl vancomycin (ΔNCBPV) (3 μg/mL)-treated *S*. *pyogenes* (**C**) (n = 3). (**A** and **C**) Typical fluorescent images and cell size distribution of phenotypes A–D′. Images: PG (growing peptidoglycan stained using the sortase method), Membrane (Nile red staining), DNA (DAPI), and Overlay (overlay of PG and Membrane images); scale bar: 2 μm. Blue bars in histograms represent the cell size distribution of all cells. Orange bars in histograms represent the size distribution cells with the indicated phenotypes. Statistical analysis regarding the histograms is available in Supplementary Table S6. (**B**) Schematic drawing and labeled PG image of phenotype D′.

Next, we investigated the effects of CBPV on *S*. *pyogenes*. The cells exhibited an axially elongated morphology (phenotype D′) that was not observed in vancomycin-treated cells. This implies distinct molecular modes of inhibition of CBPV from vancomycin (Fig. 7A). CBPV-treated cells were distributed into phenotypes A (20 ± 1%), B (58 ± 6%), C (9 ± 2%), and D′ (13 ± 4%) in three independent experiments. Phenotype D′ had multiple division sites in axially elongated cells similar to that of the related phenotype D (see the data for flavomycin-treated cells, Fig. 3). Phenotype D cells had multiple completed septa in cells. Phenotype D′ cells were different from phenotype-D cells, because they had multiple peripheral growth sites but rarely exhibited completed septa (Fig. 7B).

Only 4% of the CBPV-treated cells with multiple division sites (n = 55) possessed one or more completed septa, versus 98% (n = 114) of flavomycin-treated cells. This marked difference probably indicates the difference of antibacterial mechanisms for these two antibiotics, namely that flavomycin inhibited the cell separation step of the cell cycle, whereas CBPV inhibited the septal growth stage. This idea may also be supported by the fact that an extremely small population of cells remained at septal synthesis (phenotype C) during CBPV treatment. The cause of this potential selectivity among peripheral and septal synthesis is unclear at this stage. Collectively, vancomycin and CBPV have distinct in the modes of cell wall inhibition.

Subsequently, we focus on the derivative ΔNCBPV (Fig. 6A), which suppresses *S*. *pyogenes* growth with similar potency (3 μg/mL) as vancomycin (4 μg/mL). ΔNCBPV is a derivative of CBPV that lacks an *N*-methyl-leucyl residue at its terminus. Many studies established that d-Ala-d-Ala-binding by vancomycin involves this *N*-methyl-leucyl residue and that des-*N*-methyl-leucyl-vancomycin totally loses its antibacterial activity against gram-positive strains. However, ΔNCBPV has reduced but strong antibacterial potency. Consequently, CBPV is likely to be a dual-mechanism inhibitor of cell wall synthesis based on its vancomycin-derived d-Ala-d-Ala-binding ability (requiring *N*-methylleucine) and the effects of the lipophilic modification at its sugar moiety. ΔNCBPV appears to be a useful compound to analyze the roles of the lipophilic modification on antibacterial activity (Fig. 7C).

We found the morphologies of ΔNCBPV-treated GAS cells were distinct from those of vancomycin-treated and CBPV-treated cells, although the morphologies of ΔNCBPV-treated GAS cells were more similar to those of CBPV-treated cells. The proportions (%) of each phenotype were 14 ± 1 (phenotype A), 45 ± 12 (phenotype B), 17 ± 8 (phenotype C), and 24 ± 3 (phenotype D′) (n = 3). The accumulation of phenotype D′ indicated that ΔNCBPV also efficiently suppressed septal synthesis but allowed peripheral synthesis. The ratio of cells with at least one completed septum among all phenotype-D cells was 52% in the ΔNCBPV-treated cells (n = 105), versus 98% (n = 114) in flavomycin-treated *S*. *pyogenes*.

A previous kinetic study using purified enzyme and radiolabeled substrate revealed that CBPV and ΔNCBPV inhibit the same transglycosylation step of peptidoglycan biosynthesis via a direct interaction with PBPs^16^. Flavomycin is also known to bind to the same transglycosylase domain of purified high-molecular-weight PBPs. Our whole-cell-based assay suggested the possibility that flavomycin may behave as a cell separation inhibitor rather than a transglycosylase inhibitor for living bacteria.

Most previous synthetic studies of vancomycin derivatives speculated that the resulting derivatives should exhibit enhanced antibacterial actions with similar mechanisms as vancomycin^17^. This is in part due to the lack of a concise, easy-to-perform assay. The results in this section clearly indicate that even a single-site modification could dramatically change the modes of action of glycopeptides, in line with previous biochemical studies^16, 18^. However, our assay may be a more concise, readily available alternative means by which to analyze such changes in actions.

## Discussion

**Figure 8:**
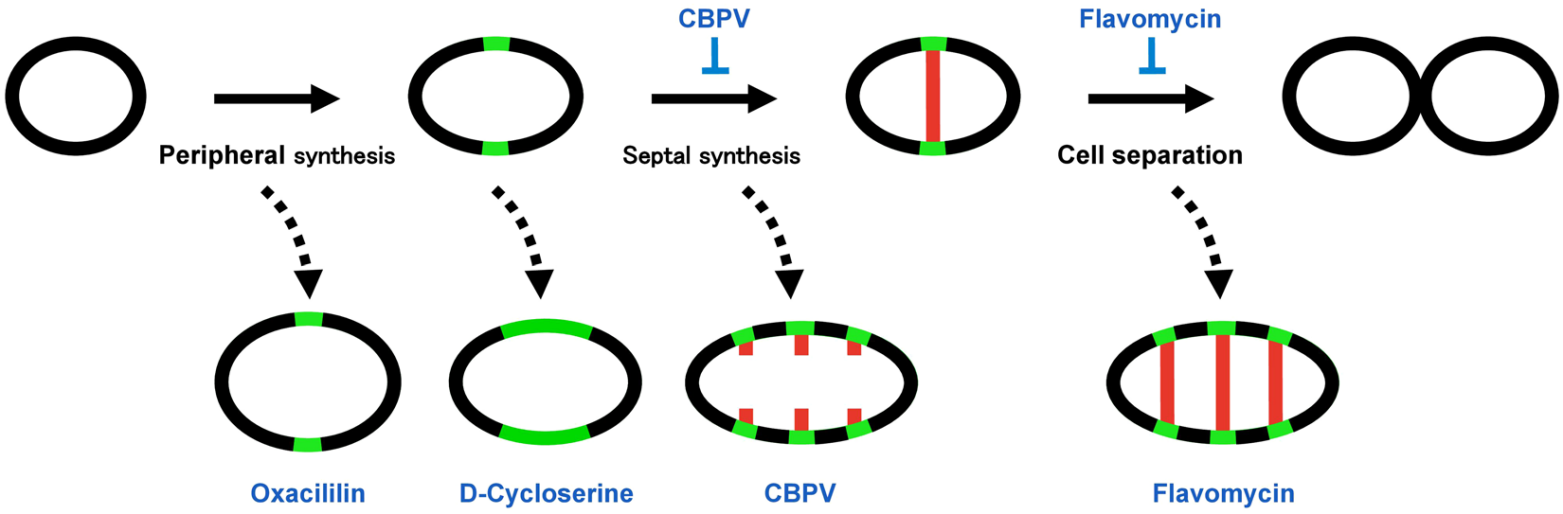
Inhibitory effects of antibiotics on *S*. *pyogenes* cell division.

In this study, we observed peripheral and septal cell wall syntheses in ovococcal *S*. *pyogenes* using “sortase-mediated” metabolic labeling for the first time. Fluorescent labeling of peripheral growth was observed in close proximity to the mid-cell division site in accordance with previous results obtained with *S*. *pneumoniae* ^19^. The fluorescent labeling of peripheral cell wall synthesis is a significant advantage of our assay because this synthesis cannot be observed by optical microscopy without labeling. This novel imaging-based assay was then applied to analyze the actions of major antibiotics (Fig. 8). Because sortase exists in most gram-positive bacteria, our technique may also be applied in bacteria other than *S*. *pyogenes*.

Metabolic labeling techniques are not limited to the sortase-based method, and are attracting significant attention in microbiology and its related fields. FDAA is also incorporated into growing bacterial cell wall^10–12^. This FDAA-based method may also be applied for imaging-based mode-of-action analyses of antibiotics in the near future. However, the incorporation of FDAA may be interfered by antibiotics, because it is mediated by PBPs^20^ that are the targets of antibiotics, including beta-lactams. Sortase is less likely to be interfered by these antibiotics, and thus, we decide to use sortase in this study.

By visualizing the growing peptidoglycan, we first illustrated that the enlarged morphology of d-cycloserine-treated cells is ascribed to the excessive peripheral synthesis. We also demonstrated that flavomycin or CBPV treatment resulted in abnormally elongated cells with multiple division sites, which may be related to a recent report of *S*. *pneumoniae* ^21^. The *S*. *pneumoniae* study demonstrated that PBPs migrate to the next division site and that they initiate peripheral synthesis before the completion of septal synthesis. Thus, it may be possible to assume that neither of these drugs suppresses the migration of PBPs or peripheral synthesis resulting in abnormally elongated cells possessing multiple division sites. Other examined antibiotics (bacitracin, ramoplanin, oxacillin, and vancomycin) displayed unique inhibitory patterns that were different from those of the aforementioned drugs.

The above intriguing observations with CBPV, a lipoglycopeptide, may support the importance of our technique in current antibacterial research. Lipoglycopeptides have recently been attracting exceptional attention because of recent approval of oritavancin, telavancin, and dalbavancin from the FDA as antibacterial agents^22^. Oritavancin and CBPV share identical lipophilic modifications in their structures. Several lipoglycopeptides are being investigated in non-/pre-clinical studies^18, 23–25^. Our assay would be useful in the medicinal chemistry of lipoglycopeptide antibiotics.

In conclusion, our imaging-based integrated protocols can offer invaluable rapid feedback to the medicinal chemists regarding newly synthesized or isolated antibacterial compounds and thus can accelerate the selection of drug leads based on both potent activity and antibacterial modes of action. These protocols may also be applicable to other pathogens.

## Materials and Methods

### Bacterial strain

*S*. *pyogenes* strain JRS4 (M6^+^ F1^+^) was grown in Todd-Hewitt broth supplemented with 0.3% yeast extract (THY) at 37 °C.

### Chemicals

Bacitracin, d-cycloserine, oxacillin, vancomycin, DAPI and Nile red stain were purchased from Wako Chemical Industries, Ltd. (Tokyo, Japan). Flavomycin and ramoplanin were obtained from Sigma-Aldrich Co. (St. Louis, MO). Van-FL stain is a product of Molecular Probes^TM^. Chlorobiphenyl vancomycin (CBPV), des-*N*-methylleucyl CBPV (ΔNCBPV), the fluorescently labeled sortase substrate FL-KLPETG-NH_2_ (FL = *N* ^ε^-fluorescein-lysine)^2^, and FDAAs^12^ (FL-d-Lys for Fig. 5A and E; tetramethylrhodamine- d-Lys for Fig. 5D) were synthesized according to the literature procedures.

### Determination of subbacteriostatic concentrations

*S*. *pyogenes* were grown in THY to an OD_600_ of 0.4 (exponential phase) at 37 °C and then added a solution of antibiotic of varying concentration. The culture mixture was further incubated for 4 h at 37 °C. Subbacteriostatic concentration in this study is the minimum dose of antibiotic that makes growth curve reach a plateau at OD_600_ of <1.0 after the 4-h incubation. In the absence of antibiotics, GAS cells grew up to OD_600_ of approximately 1.6. Subbacteriostatic concentrations were determined for each antibiotic, and mode-of-action analyses were performed at these concentrations (Figs 2, 3, 4, 5, 6 and 7).

### Live cell labeling with sortase A substrate in the presence of antibiotics

Bacterial cells grown in THY to an OD_600_ of 0.35–0.4 (exponential phase) at 37 °C were exposed to antibiotics and incubated for 1 h, and 100-μL aliquots of culture mixture were taken and added to a solution of sortase substrate (FL-KLPETG-NH_2_, final concentration = 1 mM). After incubation for 2.5 h at 37 °C in the dark, Nile Red (10 μg/mL) and DAPI (1 μg/mL) were added. The mixture was allowed to stand for 30 min and then centrifuged (12,000 rpm, 2 min, 4 °C). The supernatant was removed, and the pellet was washed thrice with PBS buffer to remove unreacted sortase substrate. Cells were re-suspended in PBS buffer, mounted on a glass slide, and examined by fluorescent microscopy. Images were analyzed manually using ImageJ software (ver. 1.50). Sets of axial- and equatorial-length were also recorded for each cell. At least 200 cells were analyzed in each analysis. All experiments were performed at least thrice, and representative results are shown.

### Imaging of peripheral growths in d-cycloserine/oxacillin-treated cells

Bacterial cells grown in THY to an OD_600_ of 0.2 (exponential phase) at 37 °C, and 100-μL aliquots of culture mixture were taken (Fig. 5). FDAA solution (final concentration = 1 mM; FL-d-Lys for Fig. 5A and E, tetramethylrhodamine- d-Lys for Fig. 5D) was added, and the mixture was incubated for 1 h at 37 °C in the dark. The mixture was centrifuged (12,000 rpm, 2 min, 4 °C), the supernatant was removed, and the pellet was washed with PBS buffer thrice to remove unreacted FDAA. Cells were re-suspended in 100-μL THY and incubated at 37 °C in the dark with antibiotics for 2.5 h. A reagent (10 μg/mL Nile Red for Fig. 5A and E; 1 μg/mL Van-FL for Fig. 5D) was then added to the mixture, which was allowed to stand for 30 min before centrifugation (12,000 rpm, 2 min, 4 °C). The supernatant was removed, and the pellet was washed thrice with PBS buffer. Cells were re-suspended in PBS buffer, mounted on a glass slide, and examined by fluorescent microscopy.

The datasets generated during and/or analysed during the current study are available from the corresponding author on reasonable request.

## Electronic supplementary material

Supplementary information
