# Supplementary material for: Deciphering the mode of action of cell wall-inhibiting antibiotics using metabolic labeling of growing peptidoglycan in Streptococcus pyogenes
Source: Sci Rep. 2017 Apr 25;7:1129. doi: 10.1038/s41598-017-01267-5 (PMC5430839; doi:10.1038/s41598-017-01267-5)
Supplement: Supplementary file 1 — Supplementary information [file 41598_2017_1267_MOESM1_ESM.pdf]

Supplementary Materials for

Deciphering the mode of action of cell wall-inhibiting antibiotics using metabolic labeling of growing peptidoglycan in *Streptococcus pyogenes*

Atsushi Sugimoto [†], Asuka Maeda [†], Kaori Itto, Hirokazu Arimoto *

Graduate School of Life Sciences, Tohoku University, Sendai, Miyagi, Japan

Running Head: Mode of action analysis using metabolic labeling

*Address correspondence to Hirokazu Arimoto, arimoto@tohoku.ac.jp

† These authors contributed equally to this work.

Supplementary Table S1. Statistical analysis data for histograms in Figure 1C.

without antibiotics

Axial length

length (μm)	% of cells				
	all	A	B	C	D
0.5	0 ± 0	0 ± 0	0 ± 0	0 ± 0	0 ± 0
0.7	7 ± 1	6 ± 1	0 ± 0	0 ± 0	0 ± 0
0.9	25 ± 0	18 ± 2	7 ± 1	0 ± 0	0 ± 0
1.1	32 ± 1	10 ± 1	21 ± 2	1 ± 1	0 ± 0
1.3	20 ± 2	2 ± 1	14 ± 1	4 ± 1	0 ± 0
1.5	10 ± 2	0 ± 0	4 ± 1	5 ± 1	0 ± 0
1.7	5 ± 1	0 ± 0	1 ± 0	4 ± 1	0 ± 0
1.9	1 ± 0	0 ± 0	0 ± 0	1 ± 0	0 ± 0
2.1	0 ± 0	0 ± 0	0 ± 0	0 ± 0	0 ± 0
2.3	0 ± 0	0 ± 0	0 ± 0	0 ± 0	0 ± 0
2.5	0 ± 0	0 ± 0	0 ± 0	0 ± 0	0 ± 0
2.7	0 ± 0	0 ± 0	0 ± 0	0 ± 0	0 ± 0
2.9	0 ± 0	0 ± 0	0 ± 0	0 ± 0	0 ± 0
3.1	0 ± 0	0 ± 0	0 ± 0	0 ± 0	0 ± 0

Data represent the mean ± sem (n=3).

Equatorial length

length (μm)	% of cells				
	all	A	B	C	D
0.5	0 ± 0	0 ± 0	0 ± 0	0 ± 0	0 ± 0
0.7	7 ± 2	4 ± 2	2 ± 1	1 ± 1	0 ± 0
0.9	47 ± 9	21 ± 2	20 ± 5	6 ± 2	0 ± 0
1.1	32 ± 5	10 ± 2	18 ± 2	4 ± 2	0 ± 0
1.3	8 ± 4	1 ± 0	5 ± 3	2 ± 1	0 ± 0
1.5	4 ± 3	1 ± 0	2 ± 1	2 ± 1	0 ± 0
1.7	2 ± 2	0 ± 0	0 ± 0	2 ± 2	0 ± 0
1.9	0 ± 0	0 ± 0	0 ± 0	0 ± 0	0 ± 0
2.1	0 ± 0	0 ± 0	0 ± 0	0 ± 0	0 ± 0
2.3	0 ± 0	0 ± 0	0 ± 0	0 ± 0	0 ± 0
2.5	0 ± 0	0 ± 0	0 ± 0	0 ± 0	0 ± 0
2.7	0 ± 0	0 ± 0	0 ± 0	0 ± 0	0 ± 0
2.9	0 ± 0	0 ± 0	0 ± 0	0 ± 0	0 ± 0
3.1	0 ± 0	0 ± 0	0 ± 0	0 ± 0	0 ± 0

Data represent the mean ± sem (n=3).

Supplementary Table S2. Statistical analysis data for histograms in Figure 2.

Bacitracin (Figure 2A)

Axial length

length (µm)	% of cells				
	all	A	B	C	D
0.5	1 ± 1	1 ± 0	0 ± 0	0 ± 0	0 ± 0
0.7	13 ± 2	9 ± 1	4 ± 1	0 ± 0	0 ± 0
0.9	37 ± 3	17 ± 4	18 ± 1	2 ± 0	0 ± 0
1.1	29 ± 2	5 ± 1	19 ± 3	4 ± 0	0 ± 0
1.3	15 ± 1	1 ± 0	8 ± 0	5 ± 1	0 ± 0
1.5	3 ± 1	1 ± 0	1 ± 0	1 ± 0	0 ± 0
1.7	1 ± 0	0 ± 0	1 ± 0	0 ± 0	0 ± 0
1.9	0 ± 0	0 ± 0	0 ± 0	0 ± 0	0 ± 0
2.1	0 ± 0	0 ± 0	0 ± 0	0 ± 0	0 ± 0
2.3	0 ± 0	0 ± 0	0 ± 0	0 ± 0	0 ± 0
2.5	0 ± 0	0 ± 0	0 ± 0	0 ± 0	0 ± 0
2.7	0 ± 0	0 ± 0	0 ± 0	0 ± 0	0 ± 0
2.9	0 ± 0	0 ± 0	0 ± 0	0 ± 0	0 ± 0
3.1	0 ± 0	0 ± 0	0 ± 0	0 ± 0	0 ± 0

Data represent the mean ± sem (n=3).

Equatorial length

length (µm)	% of cells				
	all	A	B	C	D
0.5	1 ± 1	0 ± 0	0 ± 0	1 ± 1	0 ± 0
0.7	30 ± 13	10 ± 2	16 ± 9	4 ± 2	0 ± 0
0.9	53 ± 10	20 ± 5	26 ± 5	7 ± 2	0 ± 0
1.1	15 ± 4	4 ± 2	10 ± 3	1 ± 0	0 ± 0
1.3	1 ± 1	0 ± 0	0 ± 0	0 ± 0	0 ± 0
1.5	0 ± 0	0 ± 0	0 ± 0	0 ± 0	0 ± 0
1.7	0 ± 0	0 ± 0	0 ± 0	0 ± 0	0 ± 0
1.9	0 ± 0	0 ± 0	0 ± 0	0 ± 0	0 ± 0
2.1	0 ± 0	0 ± 0	0 ± 0	0 ± 0	0 ± 0
2.3	0 ± 0	0 ± 0	0 ± 0	0 ± 0	0 ± 0
2.5	0 ± 0	0 ± 0	0 ± 0	0 ± 0	0 ± 0
2.7	0 ± 0	0 ± 0	0 ± 0	0 ± 0	0 ± 0
2.9	0 ± 0	0 ± 0	0 ± 0	0 ± 0	0 ± 0
3.1	0 ± 0	0 ± 0	0 ± 0	0 ± 0	0 ± 0

Data represent the mean ± sem (n=3).

Ramoplanin (Figure 2B)

Axial length

length (µm)	% of cells				
	all	A	B	C	D
0.5	1 ± 1	1 ± 1	0 ± 0	0 ± 0	0 ± 0
0.7	11 ± 5	9 ± 4	1 ± 1	0 ± 0	0 ± 0
0.9	25 ± 1	11 ± 1	14 ± 2	1 ± 0	0 ± 0
1.1	29 ± 3	5 ± 1	20 ± 3	4 ± 1	0 ± 0
1.3	20 ± 4	1 ± 0	10 ± 2	9 ± 1	0 ± 0
1.5	10 ± 4	0 ± 0	4 ± 1	7 ± 3	0 ± 0
1.7	1 ± 1	0 ± 0	0 ± 0	1 ± 0	0 ± 0
1.9	1 ± 1	0 ± 0	0 ± 0	1 ± 0	0 ± 0
2.1	0 ± 0	0 ± 0	0 ± 0	0 ± 0	0 ± 0
2.3	0 ± 0	0 ± 0	0 ± 0	0 ± 0	0 ± 0
2.5	0 ± 0	0 ± 0	0 ± 0	0 ± 0	0 ± 0
2.7	0 ± 0	0 ± 0	0 ± 0	0 ± 0	0 ± 0
2.9	0 ± 0	0 ± 0	0 ± 0	0 ± 0	0 ± 0
3.1	0 ± 0	0 ± 0	0 ± 0	0 ± 0	0 ± 0

Data represent the mean ± sem (n=3).

Equatorial length

length (µm)	% of cells				
	all	A	B	C	D
0.5	0 ± 0	0 ± 0	0 ± 0	0 ± 0	0 ± 0
0.7	13 ± 2	5 ± 1	3 ± 1	5 ± 1	0 ± 0
0.9	62 ± 5	17 ± 3	33 ± 4	12 ± 2	0 ± 0
1.1	23 ± 6	4 ± 2	12 ± 3	6 ± 2	0 ± 0
1.3	1 ± 0	0 ± 0	1 ± 0	0 ± 0	0 ± 0
1.5	0 ± 0	0 ± 0	0 ± 0	0 ± 0	0 ± 0
1.7	0 ± 0	0 ± 0	0 ± 0	0 ± 0	0 ± 0
1.9	0 ± 0	0 ± 0	0 ± 0	0 ± 0	0 ± 0
2.1	0 ± 0	0 ± 0	0 ± 0	0 ± 0	0 ± 0
2.3	0 ± 0	0 ± 0	0 ± 0	0 ± 0	0 ± 0
2.5	0 ± 0	0 ± 0	0 ± 0	0 ± 0	0 ± 0
2.7	0 ± 0	0 ± 0	0 ± 0	0 ± 0	0 ± 0
2.9	0 ± 0	0 ± 0	0 ± 0	0 ± 0	0 ± 0
3.1	0 ± 0	0 ± 0	0 ± 0	0 ± 0	0 ± 0

Data represent the mean ± sem (n=3).

Supplementary Table S3. Statistical analysis data for histograms in Figure 3.

Flavomycin (Figure 3B)

Axial length

length (μm)	% of cells				
	all	A	B	C	D
0.5	0 ± 0	0 ± 0	0 ± 0	0 ± 0	0 ± 0
0.7	1 ± 0	0 ± 0	0 ± 0	0 ± 0	0 ± 0
0.9	3 ± 1	1 ± 0	2 ± 1	0 ± 0	0 ± 0
1.1	6 ± 1	1 ± 0	3 ± 1	2 ± 0	0 ± 0
1.3	11 ± 3	1 ± 0	5 ± 2	4 ± 1	1 ± 0
1.5	15 ± 4	0 ± 0	6 ± 2	5 ± 1	4 ± 1
1.7	15 ± 2	0 ± 0	2 ± 0	7 ± 1	5 ± 1
1.9	16 ± 1	0 ± 0	1 ± 0	4 ± 1	11 ± 2
2.1	9 ± 2	0 ± 0	0 ± 0	1 ± 0	7 ± 2
2.3	8 ± 1	0 ± 0	0 ± 0	0 ± 0	7 ± 1
2.5	4 ± 1	0 ± 0	0 ± 0	0 ± 0	4 ± 1
2.7	4 ± 2	0 ± 0	0 ± 0	0 ± 0	4 ± 1
2.9	2 ± 1	0 ± 0	0 ± 0	0 ± 0	2 ± 1
3.1	7 ± 5	0 ± 0	0 ± 0	0 ± 0	7 ± 5

Data represent the mean ± sem (n=3).

Equatorial length

length (μm)	% of cells				
	all	A	B	C	D
0.5	0 ± 0	0 ± 0	0 ± 0	0 ± 0	0 ± 0
0.7	3 ± 1	0 ± 0	1 ± 0	0 ± 0	2 ± 1
0.9	22 ± 5	2 ± 0	4 ± 1	6 ± 2	10 ± 3
1.1	36 ± 8	1 ± 0	9 ± 3	8 ± 1	19 ± 4
1.3	22 ± 5	1 ± 0	4 ± 1	5 ± 2	12 ± 4
1.5	11 ± 6	1 ± 0	1 ± 0	2 ± 1	7 ± 5
1.7	3 ± 2	0 ± 0	0 ± 0	0 ± 0	3 ± 2
1.9	1 ± 1	0 ± 0	0 ± 0	0 ± 0	1 ± 1
2.1	0 ± 0	0 ± 0	0 ± 0	0 ± 0	0 ± 0
2.3	0 ± 0	0 ± 0	0 ± 0	0 ± 0	0 ± 0
2.5	0 ± 0	0 ± 0	0 ± 0	0 ± 0	0 ± 0
2.7	0 ± 0	0 ± 0	0 ± 0	0 ± 0	0 ± 0
2.9	0 ± 0	0 ± 0	0 ± 0	0 ± 0	0 ± 0
3.1	0 ± 0	0 ± 0	0 ± 0	0 ± 0	0 ± 0

Data represent the mean ± sem (n=3).

Supplementary Table S4. Statistical analysis data for histograms in Figure 4.

D-Cycloserine (Figure 4A)

Axial length

length	% of cells				
(μm)	all	A	B	C	D
0.5	0 ± 0	0 ± 0	0 ± 0	0 ± 0	0 ± 0
0.7	0 ± 0	0 ± 0	0 ± 0	0 ± 0	0 ± 0
0.9	4 ± 1	3 ± 1	2 ± 0	0 ± 0	0 ± 0
1.1	19 ± 3	9 ± 3	10 ± 2	0 ± 0	0 ± 0
1.3	30 ± 2	8 ± 2	22 ± 4	0 ± 0	0 ± 0
1.5	22 ± 2	4 ± 1	18 ± 0	1 ± 0	0 ± 0
1.7	13 ± 1	1 ± 0	11 ± 0	0 ± 0	1 ± 0
1.9	7 ± 2	0 ± 0	5 ± 1	0 ± 0	2 ± 0
2.1	2 ± 0	0 ± 0	1 ± 0	0 ± 0	1 ± 0
2.3	1 ± 0	0 ± 0	1 ± 0	0 ± 0	0 ± 0
2.5	0 ± 0	0 ± 0	0 ± 0	0 ± 0	0 ± 0
2.7	0 ± 0	0 ± 0	0 ± 0	0 ± 0	0 ± 0
2.9	0 ± 0	0 ± 0	0 ± 0	0 ± 0	0 ± 0
3.1	0 ± 0	0 ± 0	0 ± 0	0 ± 0	0 ± 0

Data represent the mean ± sem (n=3).

Equatorial length

length	% of cells				
(μm)	all	A	B	C	D
0.5	0 ± 0	0 ± 0	0 ± 0	0 ± 0	0 ± 0
0.7	2 ± 0	1 ± 1	1 ± 0	0 ± 0	0 ± 0
0.9	21 ± 3	6 ± 2	12 ± 3	1 ± 0	2 ± 0
1.1	42 ± 5	10 ± 1	29 ± 5	0 ± 0	2 ± 0
1.3	28 ± 4	6 ± 2	21 ± 2	1 ± 1	1 ± 0
1.5	7 ± 2	1 ± 1	5 ± 2	0 ± 0	0 ± 0
1.7	1 ± 0	0 ± 0	0 ± 0	0 ± 0	0 ± 0
1.9	0 ± 0	0 ± 0	0 ± 0	0 ± 0	0 ± 0
2.1	0 ± 0	0 ± 0	0 ± 0	0 ± 0	0 ± 0
2.3	0 ± 0	0 ± 0	0 ± 0	0 ± 0	0 ± 0
2.5	0 ± 0	0 ± 0	0 ± 0	0 ± 0	0 ± 0
2.7	0 ± 0	0 ± 0	0 ± 0	0 ± 0	0 ± 0
2.9	0 ± 0	0 ± 0	0 ± 0	0 ± 0	0 ± 0
3.1	0 ± 0	0 ± 0	0 ± 0	0 ± 0	0 ± 0

Data represent the mean ± sem (n=3).

Oxacillin (Figure 4B)

Axial length

length (μm)	% of cells				
	all	A	B	C	D
0.5	0 ± 0	0 ± 0	0 ± 0	0 ± 0	0 ± 0
0.7	1 ± 1	1 ± 0	0 ± 0	0 ± 0	0 ± 0
0.9	10 ± 3	6 ± 2	4 ± 1	0 ± 0	0 ± 0
1.1	20 ± 4	7 ± 2	12 ± 3	1 ± 0	0 ± 0
1.3	26 ± 1	3 ± 1	21 ± 2	1 ± 1	0 ± 0
1.5	19 ± 2	1 ± 0	15 ± 1	3 ± 1	1 ± 0
1.7	10 ± 2	0 ± 0	6 ± 1	3 ± 1	0 ± 0
1.9	6 ± 2	0 ± 0	2 ± 1	2 ± 1	1 ± 0
2.1	3 ± 1	0 ± 0	0 ± 0	1 ± 1	2 ± 1
2.3	2 ± 1	0 ± 0	0 ± 0	0 ± 0	2 ± 1
2.5	1 ± 1	0 ± 0	0 ± 0	0 ± 0	1 ± 1
2.7	1 ± 0	0 ± 0	0 ± 0	0 ± 0	1 ± 0
2.9	0 ± 0	0 ± 0	0 ± 0	0 ± 0	0 ± 0
3.1	0 ± 0	0 ± 0	0 ± 0	0 ± 0	0 ± 0

Data represent the mean ± sem (n=3).

Equatorial length

length (μm)	% of cells				
	all	A	B	C	D
0.5	0 ± 0	0 ± 0	0 ± 0	0 ± 0	0 ± 0
0.7	4 ± 2	1 ± 1	2 ± 1	0 ± 0	0 ± 0
0.9	21 ± 6	7 ± 1	12 ± 5	1 ± 0	1 ± 0
1.1	42 ± 8	13 ± 4	26 ± 6	1 ± 1	2 ± 1
1.3	26 ± 8	3 ± 1	18 ± 4	3 ± 2	2 ± 1
1.5	6 ± 5	0 ± 0	4 ± 3	2 ± 1	1 ± 0
1.7	1 ± 1	0 ± 0	0 ± 0	0 ± 0	0 ± 0
1.9	1 ± 0	0 ± 0	0 ± 0	0 ± 0	0 ± 0
2.1	0 ± 0	0 ± 0	0 ± 0	0 ± 0	0 ± 0
2.3	0 ± 0	0 ± 0	0 ± 0	0 ± 0	0 ± 0
2.5	0 ± 0	0 ± 0	0 ± 0	0 ± 0	0 ± 0
2.7	0 ± 0	0 ± 0	0 ± 0	0 ± 0	0 ± 0
2.9	0 ± 0	0 ± 0	0 ± 0	0 ± 0	0 ± 0
3.1	0 ± 0	0 ± 0	0 ± 0	0 ± 0	0 ± 0

Data represent the mean ± sem (n=3).

Supplementary Table S5. Statistical analysis data for histograms in Figure 6.

Vancomycin (Figure 6B)

Axial length

length (μm)	% of cells				
	all	A	B	C	D
0.5	0 ± 0	0 ± 0	0 ± 0	0 ± 0	0 ± 0
0.7	15 ± 5	11 ± 4	4 ± 2	0 ± 0	0 ± 0
0.9	28 ± 2	13 ± 2	14 ± 3	1 ± 0	0 ± 0
1.1	29 ± 2	8 ± 3	19 ± 1	3 ± 1	0 ± 0
1.3	18 ± 2	2 ± 1	9 ± 2	7 ± 1	0 ± 0
1.5	8 ± 2	0 ± 0	3 ± 1	5 ± 0	0 ± 0
1.7	2 ± 1	0 ± 0	0 ± 0	2 ± 1	0 ± 0
1.9	0 ± 0	0 ± 0	0 ± 0	0 ± 0	0 ± 0
2.1	0 ± 0	0 ± 0	0 ± 0	0 ± 0	0 ± 0
2.3	0 ± 0	0 ± 0	0 ± 0	0 ± 0	0 ± 0
2.5	0 ± 0	0 ± 0	0 ± 0	0 ± 0	0 ± 0
2.7	0 ± 0	0 ± 0	0 ± 0	0 ± 0	0 ± 0
2.9	0 ± 0	0 ± 0	0 ± 0	0 ± 0	0 ± 0
3.1	0 ± 0	0 ± 0	0 ± 0	0 ± 0	0 ± 0

Data represent the mean ± sem (n=3).

Equatorial length

length (μm)	% of cells				
	all	A	B	C	D
0.5	0 ± 0	0 ± 0	0 ± 0	0 ± 0	0 ± 0
0.7	25 ± 8	10 ± 2	11 ± 4	5 ± 2	0 ± 0
0.9	56 ± 2	19 ± 2	27 ± 4	10 ± 1	0 ± 0
1.1	17 ± 9	5 ± 3	9 ± 4	2 ± 1	0 ± 0
1.3	2 ± 1	0 ± 0	2 ± 1	0 ± 0	0 ± 0
1.5	0 ± 0	0 ± 0	0 ± 0	0 ± 0	0 ± 0
1.7	0 ± 0	0 ± 0	0 ± 0	0 ± 0	0 ± 0
1.9	0 ± 0	0 ± 0	0 ± 0	0 ± 0	0 ± 0
2.1	0 ± 0	0 ± 0	0 ± 0	0 ± 0	0 ± 0
2.3	0 ± 0	0 ± 0	0 ± 0	0 ± 0	0 ± 0
2.5	0 ± 0	0 ± 0	0 ± 0	0 ± 0	0 ± 0
2.7	0 ± 0	0 ± 0	0 ± 0	0 ± 0	0 ± 0
2.9	0 ± 0	0 ± 0	0 ± 0	0 ± 0	0 ± 0
3.1	0 ± 0	0 ± 0	0 ± 0	0 ± 0	0 ± 0

Data represent the mean ± sem (n=3).

Supplementary Table S6. Statistical analysis data for histograms in Figure 7.

CBPV (Figure 7A)

Axial length

length (μm)	% of cells				
	all	A	B	C	D
0.5	0 ± 0	0 ± 0	0 ± 0	0 ± 0	0 ± 0
0.7	2 ± 1	1 ± 0	0 ± 0	0 ± 0	0 ± 0
0.9	16 ± 3	7 ± 1	8 ± 2	1 ± 0	0 ± 0
1.1	29 ± 5	7 ± 1	20 ± 4	1 ± 1	0 ± 0
1.3	19 ± 3	2 ± 1	15 ± 3	2 ± 1	0 ± 0
1.5	12 ± 3	1 ± 0	7 ± 2	3 ± 0	0 ± 0
1.7	9 ± 2	0 ± 0	5 ± 2	2 ± 1	1 ± 1
1.9	5 ± 1	0 ± 0	2 ± 1	1 ± 0	2 ± 0
2.1	3 ± 1	0 ± 0	0 ± 0	1 ± 0	2 ± 0
2.3	2 ± 1	0 ± 0	0 ± 0	0 ± 0	2 ± 1
2.5	1 ± 0	0 ± 0	0 ± 0	0 ± 0	1 ± 0
2.7	1 ± 0	0 ± 0	0 ± 0	0 ± 0	1 ± 0
2.9	1 ± 1	0 ± 0	0 ± 0	0 ± 0	1 ± 1
3.1	1 ± 1	0 ± 0	0 ± 0	0 ± 0	1 ± 1

Data represent the mean ± sem (n=3).

Equatorial length

length (μm)	% of cells				
	all	A	B	C	D
0.5	0 ± 0	0 ± 0	0 ± 0	0 ± 0	0 ± 0
0.7	9 ± 3	2 ± 0	4 ± 2	1 ± 1	1 ± 1
0.9	48 ± 6	11 ± 2	28 ± 6	4 ± 1	5 ± 1
1.1	36 ± 6	5 ± 1	23 ± 4	3 ± 1	4 ± 3
1.3	6 ± 2	1 ± 0	3 ± 1	1 ± 1	1 ± 1
1.5	1 ± 0	0 ± 0	0 ± 0	0 ± 0	0 ± 0
1.7	0 ± 0	0 ± 0	0 ± 0	0 ± 0	0 ± 0
1.9	0 ± 0	0 ± 0	0 ± 0	0 ± 0	0 ± 0
2.1	0 ± 0	0 ± 0	0 ± 0	0 ± 0	0 ± 0
2.3	0 ± 0	0 ± 0	0 ± 0	0 ± 0	0 ± 0
2.5	0 ± 0	0 ± 0	0 ± 0	0 ± 0	0 ± 0
2.7	0 ± 0	0 ± 0	0 ± 0	0 ± 0	0 ± 0
2.9	0 ± 0	0 ± 0	0 ± 0	0 ± 0	0 ± 0
3.1	0 ± 0	0 ± 0	0 ± 0	0 ± 0	0 ± 0

Data represent the mean ± sem (n=3).

ΔNCBPV (Figure 7C)

Axial length

length (μm)	% of cells				
	all	A	B	C	D
0.5	0 ± 0	0 ± 0	0 ± 0	0 ± 0	0 ± 0
0.7	1 ± 0	0 ± 0	0 ± 0	0 ± 0	0 ± 0
0.9	7 ± 2	4 ± 1	3 ± 1	1 ± 0	0 ± 0
1.1	19 ± 4	6 ± 1	13 ± 4	1 ± 0	0 ± 0
1.3	20 ± 1	2 ± 1	16 ± 3	2 ± 2	1 ± 0
1.5	14 ± 1	1 ± 0	8 ± 2	4 ± 2	1 ± 1
1.7	11 ± 0	0 ± 0	7 ± 2	3 ± 2	1 ± 0
1.9	9 ± 1	0 ± 0	2 ± 0	3 ± 1	4 ± 1
2.1	6 ± 0	0 ± 0	1 ± 0	1 ± 1	5 ± 1
2.3	4 ± 1	0 ± 0	0 ± 0	0 ± 0	3 ± 1
2.5	3 ± 1	0 ± 0	0 ± 0	0 ± 0	3 ± 1
2.7	2 ± 1	0 ± 0	0 ± 0	0 ± 0	2 ± 1
2.9	1 ± 1	0 ± 0	0 ± 0	0 ± 0	1 ± 1
3.1	3 ± 2	0 ± 0	0 ± 0	0 ± 0	3 ± 2

Data represent the mean ± sem (n=3).

Equatorial length

length (μm)	% of cells				
	all	A	B	C	D
0.5	1 ± 0	0 ± 0	0 ± 0	0 ± 0	0 ± 0
0.7	9 ± 4	2 ± 1	2 ± 2	1 ± 1	3 ± 2
0.9	34 ± 9	6 ± 1	20 ± 7	3 ± 1	5 ± 2
1.1	30 ± 3	4 ± 1	18 ± 3	5 ± 3	4 ± 1
1.3	17 ± 7	2 ± 1	5 ± 2	5 ± 3	5 ± 2
1.5	6 ± 3	0 ± 0	3 ± 1	1 ± 1	2 ± 1
1.7	3 ± 2	0 ± 0	0 ± 0	0 ± 0	2 ± 2
1.9	1 ± 1	0 ± 0	0 ± 0	0 ± 0	1 ± 1
2.1	0 ± 0	0 ± 0	0 ± 0	0 ± 0	0 ± 0
2.3	0 ± 0	0 ± 0	0 ± 0	0 ± 0	0 ± 0
2.5	0 ± 0	0 ± 0	0 ± 0	0 ± 0	0 ± 0
2.7	0 ± 0	0 ± 0	0 ± 0	0 ± 0	0 ± 0
2.9	0 ± 0	0 ± 0	0 ± 0	0 ± 0	0 ± 0
3.1	0 ± 0	0 ± 0	0 ± 0	0 ± 0	0 ± 0

Data represent the mean ± sem (n=3).
